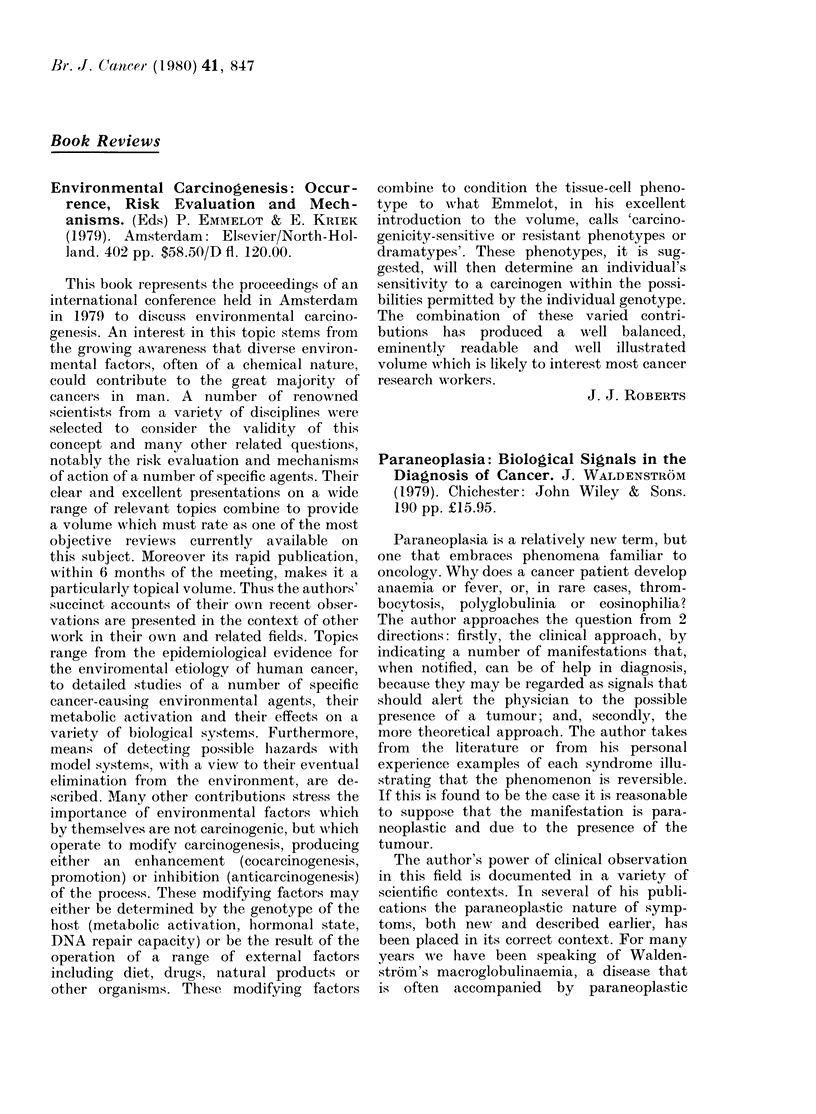# Environmental Carcinogenesis: Occurrence, Risk Evaluation and Mechanisms

**Published:** 1980-05

**Authors:** J. J. Roberts


*Br. J. Cancer* (1980) **41**, 847

### *Book Reviews*

**Environmental Carcinogenesis: Occurrence, Risk Evaluation and Mechanisms.** (Eds) P. EMMELOT & E. KRIEK (1979). Amsterdam: Elsevier/North-Holland. 402 pp. $58.50/D fl. 120.00.

This book represents the proceedings of an international conference held in Amsterdam in 1979 to discuss environmental carcinogenesis. An interest in this topic stems from the growing awareness that diverse environmental factors, often of a chemical nature, could contribute to the great majority of cancers in man. A number of renowned scientists from a variety of disciplines were selected to consider the validity of this concept and many other related questions, notably the risk evaluation and mechanisms of action of a number of specific agents. Their clear and excellent presentations on a wide range of relevant topics combine to provide a volume which must rate as one of the most objective reviews currently available on this subject. Moreover its rapid publication, within 6 months of the meeting, makes it a particularly topical volume. Thus the authors' succinct accounts of their own recent observations are presented in the context of other work in their own and related fields. Topics range from the epidemiological evidence for the enviromental etiology of human cancer, to detailed studies of a number of specific cancer-causing environmental agents, their metabolic activation and their effects on a variety of biological systems. Furthermore, means of detecting possible hazards with model systems, with a view to their eventual elimination from the environment, are described. Many other contributions stress the importance of environmental factors which by themselves are not carcinogenic, but which operate to modify carcinogenesis, producing either an enhancement (cocarcinogenesis, promotion) or inhibition (anticarcinogenesis) of the process. These modifying factors may either be determined by the genotype of the host (metabolic activation, hormonal state, DNA repair capacity) or be the result of the operation of a range of external factors including diet, drugs, natural products or other organisms. These modifying factors combine to condition the tissue-cell phenotype to what Emmelot, in his excellent introduction to the volume, calls 'carcinogenicity-sensitive or resistant phenotypes or dramatypes'. These phenotypes, it is suggested, will then determine an individual's sensitivity to a carcinogen within the possibilities permitted by the individual genotype. The combination of these varied contributions has produced a well balanced, eminently readable and well illustrated volume which is likely to interest most cancer research workers.

J. J. ROBERTS